# The Emerging Role of Cardiac Magnetic Resonance Imaging in the Evaluation of Patients with HFpEF

**DOI:** 10.1007/s11897-018-0372-1

**Published:** 2018-02-05

**Authors:** Jessica Webb, Lauren Fovargue, Kristin Tøndel, Bradley Porter, Benjamin Sieniewicz, Justin Gould, Christopher Aldo Rinaldi, Tevfik Ismail, Amedeo Chiribiri, Gerald Carr-White

**Affiliations:** 1 Department of Cardiology, Guy’s and St Thomas’ NHS Foundation Trust, St Thomas’ Hospital, Westminster Bridge Road, London, SE1 7EH UK; 2 Division of Imaging Sciences and Biomedical Engineering, King’s College London, London, SE1 7EH UK; 3 Division for Methods, Data Collection and Methods, Statistics Norway, Oslo, Norway; 4 Department of Mathematical Sciences and Technology, Norwegian University of Life Sciences, Ås, Norway

**Keywords:** Cardiac magnetic resonance (CMR), Diastolic dysfunction, Left ventricular hypertrophy, Left atrial enlargement

## Abstract

**Purpose of Review:**

To give an update on the emerging role of cardiac magnetic resonance imaging in the evaluation of patients with heart failure with preserved ejection fraction (HFpEF). This is important as the diagnosis of HFpEF remains challenging and cardiac imaging is pivotal in establishing the function of the heart and whether there is evidence of structural heart disease or diastolic dysfunction. Echocardiography is widely available, although the gold standard in quantifying heart function is cardiac magnetic resonance (CMR) imaging.

**Recent Findings:**

This review includes the recently updated 2016 European Society of Cardiology guidelines on diagnosing HFpEF that define the central role of imaging in identifying patients with HFpEF. Moreover, it includes the pathophysiology in HFpEF, how CMR works, and details current CMR techniques used to assess structural heart disease and diastolic function. Furthermore, it highlights promising research techniques that over the next few years may become more used in identifying these patients.

**Summary:**

CMR has an emerging role in establishing the diagnosis of HFpEF by measuring the left ventricular ejection fraction (LVEF) and evidence of structural heart disease and diastolic dysfunction.

## Introduction

The diagnosis of heart failure with preserved ejection fraction (HFpEF) remains challenging as clinical symptoms and signs are nonspecific [1]. Cardiac imaging is pivotal as the recently updated European Society of Cardiology (ESC) guidelines defined the diagnostic criteria for HFpEF as a left ventricular ejection fraction (LVEF) that is preserved with either evidence of diastolic dysfunction or structural heart disease, typical symptoms and signs of heart failure (HF), and raised natriuretic peptides [2••]. Practically, echocardiography is widely used with up-to-date recommendations for assessment of diastolic function [3], although these are indirect assessments of LV filling and lack characterization of myocardial tissue. Cardiac magnetic resonance (CMR) imaging represents the gold standard in quantification of LVEF [4] and is increasingly used in the assessment of HF due to its unique, precise, noninvasive phenotypic characterization with high reproducibility and sufficient spatial and temporal resolution [5]. The role of CMR in characterizing diastolic function has not been fully established clinically, although it is a class I indication for a CMR in HF patients with poor acoustic windows, partly due to the need for additional sequence acquisitions and time-consuming image analysis and post-processing. This review evaluates the pathophysiology of HFpEF, how CMR works, current CMR techniques used to assess diastolic function, and highlights promising research techniques.

### Pathophysiology of HFpEF

The exact pathophysiology of HFpEF remains uncertain and is likely due to diastolic dysfunction, impaired systolic function on exercise, abnormal ventricular-arterial coupling, inflammation and endothelial dysfunction, chronotropic incompetence, altered myocardial energetics and peripheral skeletal muscle metabolism and perfusion, and pulmonary hypertension and renal insufficiency [6••]. Only recently has it been convincingly demonstrated that HFpEF represents more than a sum of all its comorbidities and is a condition in its own right [7]. Despite variations in pathophysiology, impaired diastolic ventricular filling is consistently reported across all patients, caused by increased stiffness of the left ventricle, impaired atrial-ventricular conduction of blood, and decreased relaxation capability of the myocytes [6••, 8].

### What Is Diastolic Dysfunction?

Diastole is the time taken for the left ventricle to fill between the closure of the aortic valve and the closure of the mitral valve. During this time, blood flows from the atria to the ventricles due to the pressure difference between the chambers with atrial contraction contributing to the final part of ventricular filling. Diastole is an active, ATP-consuming myocardial process. LV filling and the transmitral pressure gradient are determined by the LV and atrial geometry, loading conditions, viscoelastic properties, pericardial restraint, valve function, heart rate, and temporal synchronization of the atrial and ventricular contraction.

**Fig. 1:**
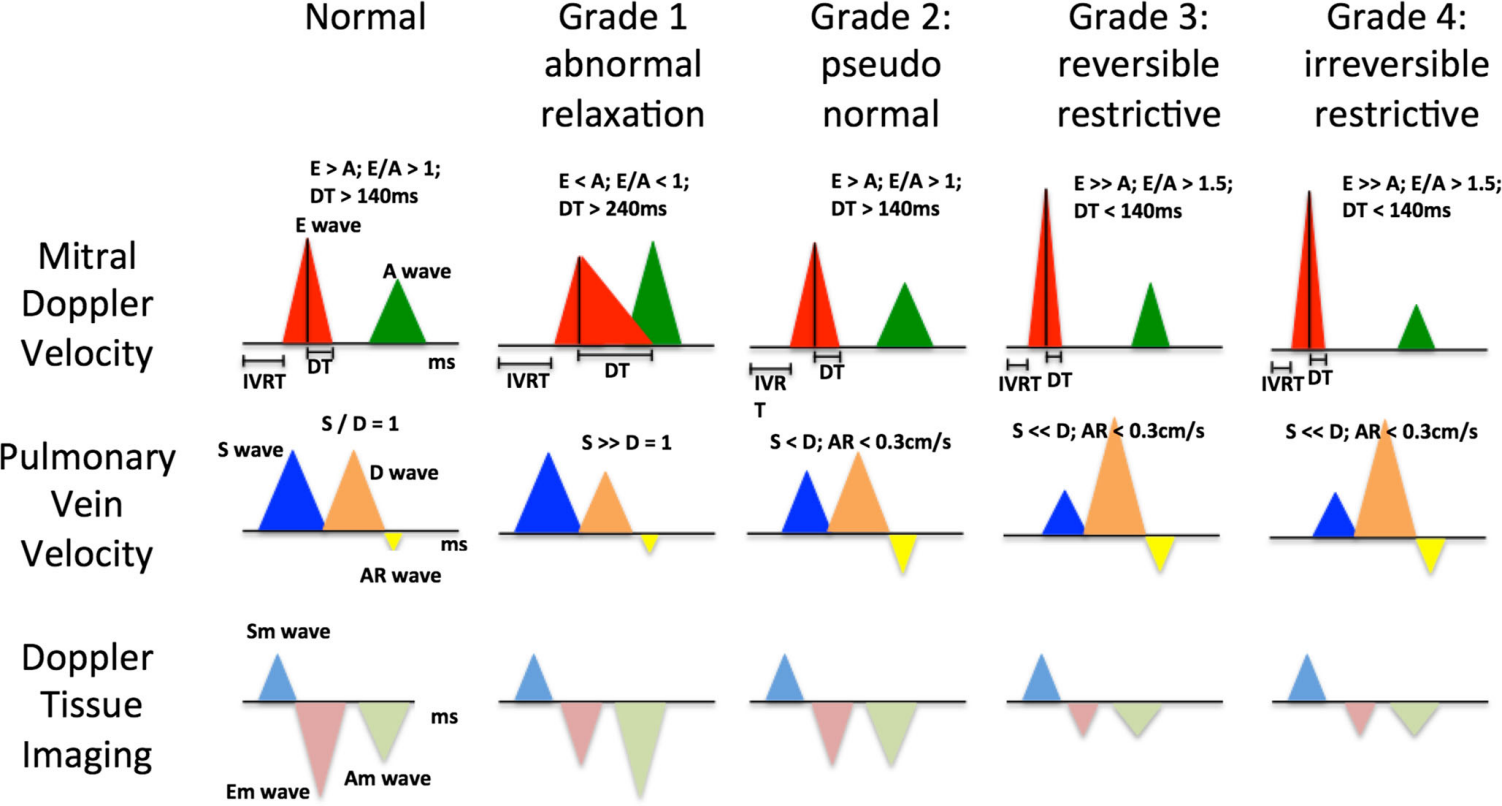
Classification of diastolic dysfunction

Diastolic dysfunction is classified into four phases [9], Fig. 1. In grade 1 diastolic dysfunction, the proportion of LV filling occurring in the early phase of diastole is reduced. Pressure in the left atrium increases as the diastolic function reduces further (grade 2). Grade 3 dysfunction occurs when there is restrictive filling due to impaired relaxation with elevated filling pressure and impaired LV compliance. With the Valsalva maneuver, the impaired filling can be reversed. Grade 4 diastolic dysfunction represents irreversible restrictive filling.

### How CMR Works

CMR uses a magnetic field, approximately 30,000 times stronger than the earth’s magnetic field, to align the nuclear magnetization of hydrogen atoms that are abundant in the human body. Image contrast is generated by the hydrogen nuclei being intermittently excited by radiofrequency pulses, resulting in longitudinal and transverse relaxation times that are characteristic for different tissues. The CMR sequence details the instructions for the radiofrequency pulses, timed data acquisitions, and magnetic gradient field switches. Spin echo sequences are mainly used for anatomic imaging and tissue characterization, whereas gradient echo sequences are used to acquire moving images. To prevent artifacts from cardiac and respiratory motion, images are gated to the R wave of the electrocardiogram and acquired in end-expiratory breath hold. Intravenous gadolinium chelated contrast agents are used to identify areas of scar or fibrosis, as a lengthened washout indicates reduced functional capillary density in the irreversibly injured myocardium.

### Current CMR Measurements to Assess Diastolic Dysfunction

**Fig. 2:**
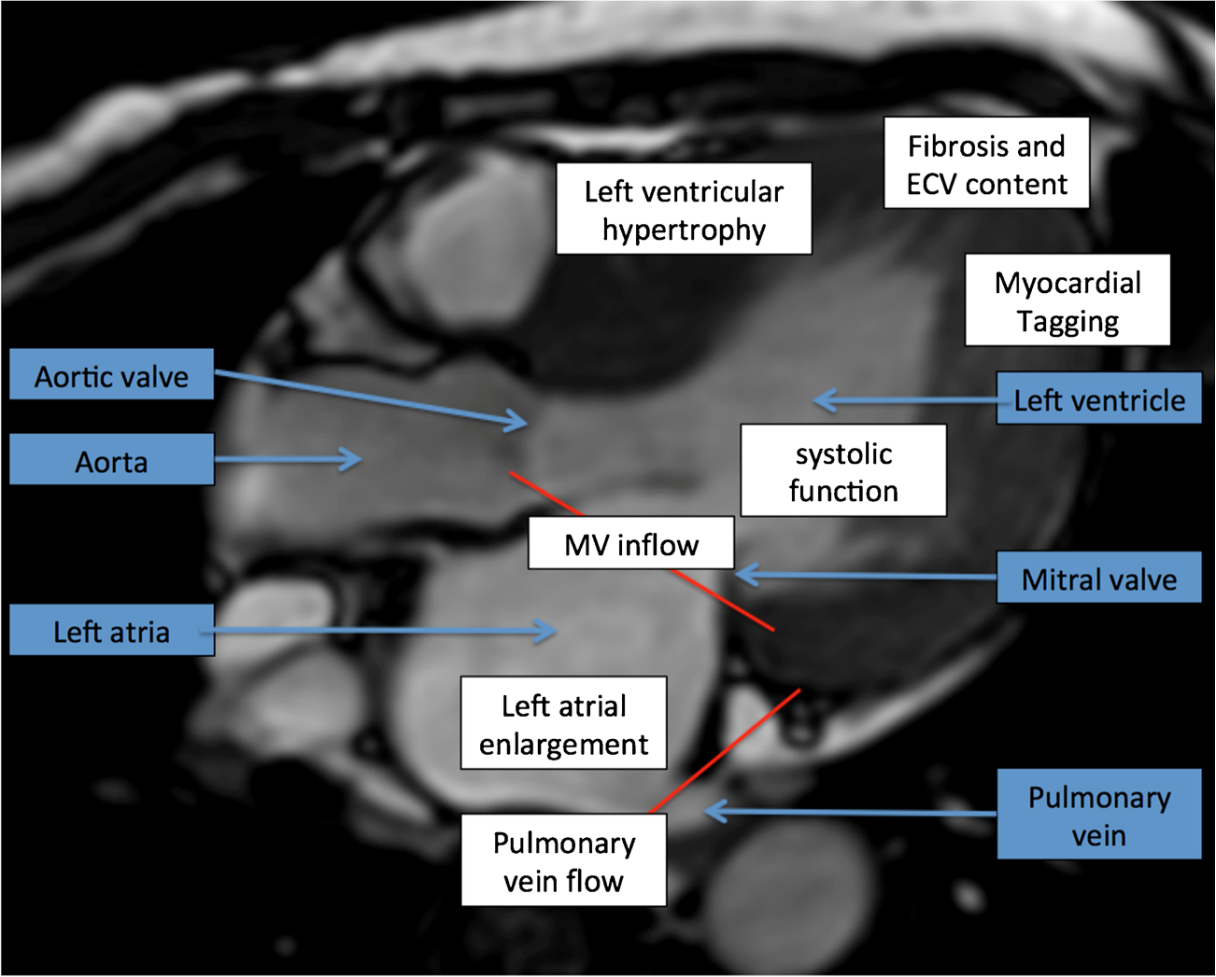
Anatomy in blue, abnormalities in HFpEF in white

In assessing a patient with HF symptoms, the LVEF first needs to be calculated. The current techniques to assess diastolic dysfunction include measuring left atrial enlargement (LAE), left ventricular hypertrophy (LVH), mitral inflow pattern, pulmonary vein assessment, LV time volume relations, LV myocardial tagging, flow propagation velocity, and calculating T1/myocardial fibrosis, Fig. 2.

### Left Atrial Size

Left atrial (LA) size, volume, and wall mass can be accurately measured by CMR, and Simpson’s volumetric method has always been considered the gold standard for measuring atrial volumes [9]. LA volume can be measured accurately from CMR imaging using the biplane area-length (BAL) technique 2 and 4 chamber images with the following equation: LA volume (ml) = (0.85 × A_2C_ × A_4C_)/L (where A2C and A4C are the LA areas on the 2 chamber and 4 chamber views, and L is the shorter length of the LA, from either the 2 or 4 chamber). Recent work in patients with HFpEF has shown that the CMR BAL technique is as accurate as CMR volumetric Simpson’s method, with echocardiography BAL technique less accurate than CMR volumetric assessment particularly with LA dilatation and in patients with atrial fibrillation [10]. However, the BAL techniques were not compared for echocardiography and CMR. The size of the atrium varies during the cardiac cycle but only maximal LA size is typically reported clinically. Body size is a major determinant of atrial size with little variation noted with gender [11].

The left atrium (LA) has three functions: during ventricular systole, it acts as a reservoir receiving blood from the pulmonary veins and storing energy in the form of pressure; during early diastole, it acts as a conduit for transfer of blood into the left ventricle via a pressure gradient and finally, it has a contractile function, augmenting the LV stroke volume. In patients with normal diastolic function, the relative contribution of the reservoir, conduit, and contractile function of the atria to the LV filling is approximately 40, 35, and 25%, respectively [12]. With abnormal LV relaxation, the relative contribution of atrial reservoir and contractile function increases, and conduit function decreases. The atria enlarge in response to both pressure and volume overload. Pressure overload is usually secondary to increased atrial afterload, either mitral valve disease or LV dysfunction, and is uniformly accompanied by abnormal myocyte relaxation. Volume overload, however, caused by athletic hearts, anemia, and mitral regurgitation can cause atrial enlargement and is normally associated with normal ventricular myocardial relaxation.

Atrial size is known to be prognostic; more than 32 ml/m^2^ is associated with increased incidence of heart failure independent of age, LV hypertrophy, diabetes, hypertension, myocardial infarction of mitral inflow velocities [13]. However there is little evidence on the diagnostic and prognostic value of left atrial volume in HFpEF patients [14], although recent evidence suggests that LA function (measured by ejection fraction) has been shown to be associated with mortality outcomes [15] and LAE was associated with the presence of dyspnea [16].

### Left Ventricular Hypertrophy

The Framingham Study reported that increased left ventricular (LV) mass is associated with a significant excess of cardiovascular mortality and morbidity, independent of hypertension of the presence of coronary artery disease [17, 18]. Left ventricular hypertrophy (LVH) is the most common structural abnormality in patients with HFpEF. It is the response of myocytes to various stimuli leading to myocyte hypertrophy, such as increased mechanical load, neurohumoral activation, and cytokines associated with arterial hypertension, diabetes, chronic renal impairment, and other comorbidities [19].

Practically, LVH can be considered as concentric remodeling (enlarged heart with normal relative wall thickness), concentric hypertrophy (increased relative wall thickness with normal LV diameter), or eccentric remodeling (increased relative wall thickness with increased LV diameter). The accuracy of CMR has been validated ex vivo using post-mortem hearts [20, 21] and it is more reproducible than both M mode and 2D echocardiography [22].

### Mitral Inflow Pattern

One of the most widely applied techniques in assessing LV diastolic function is the evaluation of the transmitral inflow of pulmonary venous flows using Doppler echocardiography. These results are load-dependent and can change dramatically with only minimal changes in heart rate or preload, and so not evaluate LV relaxation directly.

Despite these limitations, phase contrast MRI is an attractive alternative to echocardiographic pulsed wave Doppler as it allows both quantitative assessment of blood velocity and optimal positioning in 3D space of the tomographic plane of interest ensuring accurate alignment and measurements. The acquisition plane is typically perpendicular to the flow direction at the position of the mitral valve at end systole. Data is retrospectively ECG gated and can be acquired either free breathing or with a breath hold. Thirty to 40 cardiac phases are reconstructed resulting in a typical effective temporal resolution of the time velocity curve of 20–30 ms.

A time-resolved acquisition with velocity encoding perpendicular to this plane results in a time velocity curve representing one average cardiac cycle. The waveform analysis results in quantification of the early (E) and atrial (A) peak filling velocities, E/A ratio, and deceleration time of the E peak filling velocity. These can be used to classify different grades of diastolic function [9, 23].

When compared to echocardiographic results, the results of phase contrast CMR results correlated 100%, although E and A velocities were found to be systematically lower. However, the CMR acquisition of velocities in a pulsatile flow phantom correlated correctly. These differences in absolute values reflect differences in the nature of acquisition and did not result in any misclassification of diastolic flow abnormalities between modalities [24]; echo data is collected over one cardiac cycle, whereas CMR data is effectively averaged overall several cycles. These acquisitions take between 20 s and 3 min.

One challenge in CMR, however, is that the acquisition plane remains fixed during the cardiac cycle and does not move with the cyclical motion of the mitral annulus. Acquisition techniques have been introduced using moving slice velocity mapping [25] and three-dimensional three-directional velocity encoding [26]. These have shown better agreement with echo Doppler when discriminating restrictive filling patterns from other grades of diastolic dysfunction [27].

### Pulmonary Vein Assessment

Phase contrast MRI can also assess pulmonary veins. Rathi et al. found that only 68% of echo cases were able to assess the pulmonary veins, compared to 100% with CMR [24]. The blood flow waveforms of the pulmonary veins provide important information on diastolic function as LV filling and compliance, left atrial preload, and contractility all influence the left atrial filling pattern. Waveform analysis of the pulmonary venous time velocity curve includes the peak systolic velocity (S), peak anterograde diastolic velocity (D), and the peak atrial reversal velocity (Ar) in late diastole. Often there are two ventricular systolic velocity peaks, S1 and S2. S1 is related to atrial relaxation and S2 to atrial stroke volume and pulse wave propagation in the pulmonary artery. For this reason, S2 should be used to determine S/D ratio as a measure of diastolic function. Just like transmitral Doppler, the sample is placed 0.5 cm into the right upper pulmonary vein and flow velocity indices are influenced by age, although changes in LV filling and compliance affect D velocity more. Ar velocity increases with age but this does not usually exceed 35 cm/s and so values above this suggest increased LV end-diastolic pressure [28]. A difference in Ar-A duration > 30 ms indicates elevated LV end-diastolic pressure [23].

### LV Time Volume Relations

The LV time-volume relation is recorded from a series of breath-held time-resolved parallel short-axis planes or a radial stack of long-axis planes. Segmenting and summating the endocardial borders results in the end-diastolic and end-systolic LV volumes. The stroke volume and systolic function can be calculated from the difference between the end-diastolic and end-systolic volumes. Early peak filling rate, time to peak filling rate, and atrial filling fraction can also be calculated from this information. Diastolic dysfunction is associated with decreased peak filling rates and increased time to peak filling rates. Despite improvements in the definition of endocardial borders and the use of automated segmentation [29], the accuracy remains too low to use in clinical practice [30] and these parameters have limited use.

### LV Myocardial Tagging

**Fig. 3:**
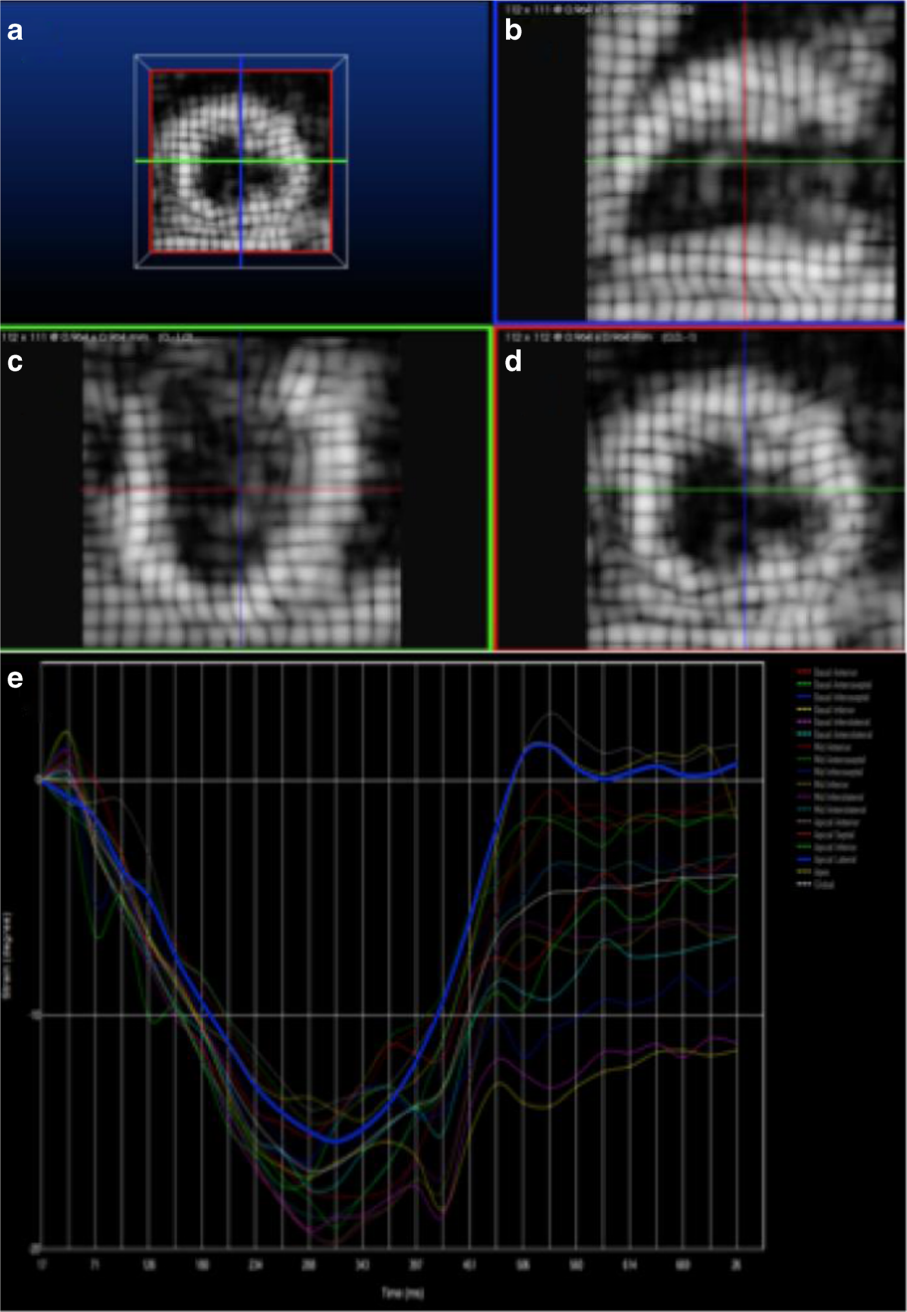
**a** Tag image of the myocardium; **b** 2 chamber view; **c** 4 chamber view; **d** short-axis image; **e** tag output for 17 segments of the LV showing deformation against time

It is possible to label or “tag” myocardium by selective saturation pulses in specific regions in planes perpendicular to the imaging plane, Fig. 3. Tag lines appear as black lines due to the saturation of signal from protons and they last for several hundred milliseconds. These lines can be tracked throughout the cardiac cycle, enabling calculations in longitudinal, radial, and circumferential directions, and are obtained every 20 milliseconds resulting in high temporal resolution. The challenges that have prevented this technique from being introduced widespread clinically are low signal to noise ratio, long acquisition times, limited availability of validated post-processing software, and that the tag lines fade during the cardiac cycle, with 3T better than 1.5T due to longer T1 and so longer persistence of the tag.

Edvardsen et al. demonstrated an association with regional diastolic dysfunction in 218 asymptomatic patients with left ventricular hypertrophy who had no evidence of clinical cardiovascular disease or LV dysfunction [31]. More recent work has been directed at feature tracking, a method of post-processing routine cine acquisitions to provide quantitative measurements of circumferential and radially directed wall strain. However, inter-study reproducibility has been shown to be poor for segmental and long-axis analyses of strain [32]. Additionally, displacement-encoded stimulated echoes (DENSE) and strain-encoded imaging (SENC) applications have both been used.

### Flow Propagation Velocity

The flow propagation velocity (Vp) is a measure of the LV suction force that has been attributed to LV relaxation. In addition to diastolic function, variables such as flow field, viscoelastic properties, inertial force, LV geometry, systolic function, mitral valve function, and LV contractile function influence intraventricular flow. A Vp > 50 cm/s in echocardiography is considered to be normal. Attempts with three-dimensional three-directional velocity encoding CMR may provide quantitative information on the intraventricular blood flow field, although long acquisition times may limit the universal uptake of this technique [33].

### Myocardial Fibrosis and Extracellular Volume Measurements

Myocardial fibrosis is a pathological increase in the myocardial collagen content caused by increased collagen synthesis in the interstitium (resulting in diffuse myocardial fibrosis) or myocyte replacement (resulting in scarring) [34]. Increased diffuse myocardial fibrosis is a major determinant of altered diastolic filling and systolic pumping function of the LV. As myocardial stiffness increases in line with the development of fibrosis, there is subsequent deterioration in both systolic and diastolic function. The pathophysiology of accumulating collagen content is diverse [34]. In HFpEF patients, there is increased collagen synthesis with elevated serum fibrotic biomarkers [35]. There has been controversy as to whether HFpEF patients have a higher collagen volume fraction (CVF). Borberly et al. looked at endomyocardial biopsies taken from HFpEF patients and demonstrated a higher CVF compared to nonHFpEF patients [36]. Although, Aoli et al. studied 172 HFpEF and HFrEF patients’ endomyocardial biopsies and found no statistically significant difference between CVF in the HFpEF and HFrEF population and that CVF did not predict outcome in HFpEF patients [37]. More recently, myocardium measured directly from HFpEF patients has been found to have a significant increase in insoluble and total collagen, as well as CVF when compared to patients with hypertension but no HFpEF [38•].

Diffuse interstitial fibrosis, a precursor for replacement fibrosis, is not detected by late gadolinium enhancement but correlates with T1 mapping, which allows a quantitative assessment of diffuse cardiac fibrosis and estimations of the extracellular matrix volume (ECV) [39–41]. Studies have validated T1 mapping and ECV estimation by CMR against histology in a variety of conditions including aortic stenosis, hypertrophic cardiomyopathy, and amyloidosis, reporting a good correlation with myocardial collagen content (*r* = 0.51~0.81) [42].

T1 mapping reflected diffuse fibrosis in 25 heart failure patients (LV ejection fraction 35 +/− 3.3%) [41]. In 2014, Mascherbauer et al. demonstrated T1 times, left atrial size, and pulmonary vascular resistance were significantly associated with cardiac events in 100 suspected HFpEF patients. The authors concluded that post-contrast T1 times are associated with prognosis and should be considered a possible HFpEF biomarker [43]. Another study looked at 62 patients with HFpEF defined as signs and symptoms of heart failure, LVEF > 45% and LV diastolic dysfunction documented by tissue Doppler echocardiography (mean septal and lateral mitral annular velocity < 8 cm/s) [44]. The authors found a significantly higher ECV in patients with both HFrEF and HFpEF, compared to patients without heart failure. In addition, ECV correlated with peak filling rate in the HFpEF population despite no correlation identified in the HFrEF population.

### Emerging CMR Imaging Techniques

Despite recent development in these techniques and the introduction of updated ESC diagnostic guidelines, no one assessment is definitive in detecting patients with HFpEF. Recent research in pulse wave velocity, MR elastography, and 4D flow assessment looks promising in these patients.

### ME Elastography

It has been hypothesized that magnetic resonance elastography (MRE) may act as a biomarker for patients with HFpEF as it uses mechanical shear waves to quantitatively assess the stiffness of tissues. Applying stress to a material results in a strain response of that tissue which can be measured. When the material is subjected to periodic shear, shear waves occur. The pair of equal forces acting in opposite directions along the two faces of the layer causes a change of shape, even though the volume remains unchanged. If the medium is elastic, then the layer will resume its original shape after the shear wave has passed. A wave is propagated as the adjacent layers undergo transient shear with equal forces acting in opposite directions, and then resume their original shape. MR elastography has been shown to map the shear stiffness of soft tissues [45]. In 2017, Arani et al. reported that 16 patients with cardiac amyloid had increased myocardial stiffness in comparison to 11 volunteers (median 11.4 kPa with range of 9.2–15.7, median 8.2 kPa with a range of 7.2–11.8, respectively, at a vibration frequency of 140 Hz) [46•].

### 4D Flow Assessment

3D phase contrast MRI, known as 4D CMR, allows volumetric flow imaging in a single acquisition. Complex blood flow patterns can be intuitively visualized as they unfold in multiple dimensions over time. Diastolic dysfunction is associated with abnormal intracardiac blood flow and it is possible to analyze quantitatively with 4D flow. This analysis can identify and monitor diastolic dysfunction. Blood transiting the left ventricle can be divided into four separate functional components: direct flow which enters and exits a single heartbeat, retained inflow which enters but does not exit, delayed ejection flow which exits on the subsequent heartbeat, and the residual volume that resides in the ventricle for at least two cardiac cycles. Investigation of these relative percentages of flow components and the kinetic energy and momentum will allow a refined analysis of the dynamic of ventricular filling and ejection. 4D flow can uniquely identify flow differences not seen with conventional hemodynamic assessment. Diminished direct flow and reduced kinetic energy of the direct flow at end diastole can be identified. As a consequence, increased workload is placed on the left ventricle to eject the same stroke volume.

### Limitations of CMR

There is no doubt that CMR offers excellent spatial resolution and is the gold standard for assessment of LV volumes and myocardial tissue characterization. However, not all departments have access to CMR imaging and the scanners are more expensive that transthoracic echocardiography, as well as taking longer. Furthermore, patient selection is critical as some patients cannot tolerate the scan due to claustrophobia (although there are limited scanners that patients are not enclosed) and patients with certain implants are not allowed in the scanner. There is a small risk of anaphylaxis due to contrast, and patients with renal impairment need reduced doses with contrast administration being contraindicated if the GFR < 15 mls/min.

## Conclusion

Cardiac MRI is fundamental in the assessment and characterization in heart failure patients. Despite challenges with long acquisition times and challenges in post-processing, CMR has a pivotal role in measuring LV systolic function and identifying left atrial enlargement and left ventricular hypertrophy, as well as other measures of diastolic function.
